# Construction and characterization of neutralizing antibody fragments for efficient access to V3 epitope

**DOI:** 10.1186/1742-4690-9-S2-P64

**Published:** 2012-09-13

**Authors:** Y Maruta

**Affiliations:** 1 Center for AIDS Research, Kumamoto University, Kumamoto, Japan

## Background

The neutralizing antibody against HIV-1 is important for the vaccine development. However, primary isolates of HIV-1 have resistance to antibody neutralization. While a neutralization epitope in V3 is exposed by conformational change of gp120 following CD4-binding, anti-V3 IgG molecules are too large to access V3-epitope. The purpose of our study is to develop Fab and scFv fragments from the anti-V3 monoclonal neutralizing antibody 5G2 for efficient access to V3-epitope.

## Methods

RNA was extracted from clonal cells producing 5G2, and cDNA was prepared by reverse transcription. Using primers for variable region of heavy and light chains, the antibody genes were amplified by PCR, and inserted into pComb3X vector. Fab and scFv were produced in the transformed E. coli by induction with isopropyl-β-D-thiogalactoside, and purified by nickel chelate chromatography.

## Results

Binding of recombinant 5G2 Fab and scFv to V3 epitope was shown by enzyme-linked immune-sorbent assay against V3 peptide. The flow cytometry analysis using cells expressing HIV-1 Env revealed the higher intensity by smaller antibody fragment, suggesting the increased accessibility of small fragment to V3 epitope. Neutralizing activity of these 5G2 fragments were observed against both primary HIV-1JR-FLwt and neutralization-sensitive variant, JR-FLL175P. In the case of JR-FLwt, scFv showed a significant improvement of neutralization.

## Conclusion

The use of smaller antibody fragments, such as scFv and Fab, may be one strategy to overcome steric constraints that confer neutralization resistance of primary isolates. We are now evaluating an effect of small antibody fragments on neutralization against various primary isolates.

